# Mechanistic insights into traditional Chinese medicine for viral pneumonia treatment: signaling pathway perspectives

**DOI:** 10.3389/fphar.2025.1577580

**Published:** 2025-11-07

**Authors:** Shihao Zheng, Tianyu Xue, Size Li, Wenying Qi, Xiaobin Zao, Peng Zhang, Fan-E Cheng, Yongan Ye, Peipei Dong

**Affiliations:** 1 Dongzhimen Hospital, Beijing University of Chinese Medicine, Beijing, China; 2 Beijing University of Chinese Medicine, Beijing, China; 3 Hebei Provincial Hospital of Traditional Chinese Medicine, Shijiazhuang, China; 4 Dongfang Hospital, Beijing University of Chinese Medicine, Beijing, China; 5 Liver Diseases Academy of Traditional Chinese Medicine, Beijing University of Chinese Medicine, Beijing, China; 6 Lianyungang TCM Hospital, Nanjing University of Chinese Medicine, Lianyungang, China

**Keywords:** traditional Chinese medicine, viral pneumonia, COVID-19, signaling pathway, pharmacology

## Abstract

Since December 2019, the World Health Organization declared COVID-19 outbreak in the World as a highly contagious respiratory disease poses a significant challenge to the world. The main symptoms of patients are cough, fever, diarrhea, etc. In addition, the COVID-19 genome has strong plasticity, and there is a risk of cross-species transmission. The use of western medicine antibiotics brings good therapeutic effects, but also accompanied by many adverse reactions of physical and mental damage. At present, TCM has achieved remarkable results in the treatment of COVID-19. In addition to enriching the cognitive theories of traditional Chinese medicine in the treatment of COVID-19, studies on the cell signal transduction mechanism of TCM in the treatment of COVID-19 have developed rapidly from the perspective of molecular biology. Through literature search, it is found that the occurrence of COVID-19 is closely related to cellular inflammatory response, immune response, apoptosis, proliferation and other physiological and pathological processes. This study systematically elucidates the molecular mechanisms by which traditional Chinese medicine treats COVID-19 by regulating key signaling pathways such as PI3K/Akt, NF-κB, JAK/STAT, and mTOR. It not only effectively alleviates COVID-19 symptoms and suppresses pulmonary inflammation but also reduces complications and drug-related adverse reactions. The integrated traditional Chinese and Western medicine model demonstrates significant synergistic effects in antiviral treatment and overall regulation. Future research should further explore the cross-mechanisms of signaling pathways, strengthen evidence-based medical validation, promote the modernization of traditional Chinese medicine, and provide safer and more effective treatment strategies for global pandemic control.

## Introduction

Viral pneumonia is an inflammation of the lungs caused by a viral infection, primarily affecting the alveoli and lung interstitium, leading to impaired gas exchange. As a common type of pneumonia, it is particularly prevalent among children, the elderly, and individuals with compromised immune systems. Since December 2019, a novel coronavirus outbreak has occurred in various parts of the world. On 12 March 2020, the World Health Organization declared Corona Virus Disease 2019 (COVID-19) a global pandemic and the world’s sixth public health emergency, which is causing a serious public health threat worldwide. COVID-19 is an infectious respiratory disease caused by severe acute respiratory syndrome coronavirus type 2 (SARS-COV-2) (Majumder and Minko, 2021).

**FIGURE 1:**
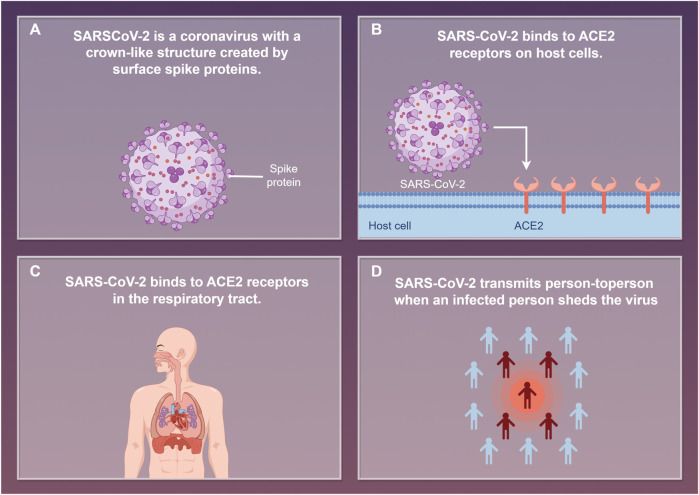
In the figure, **(A-D)** represent the transmission of SARS-COV-2.

**FIGURE 2:**
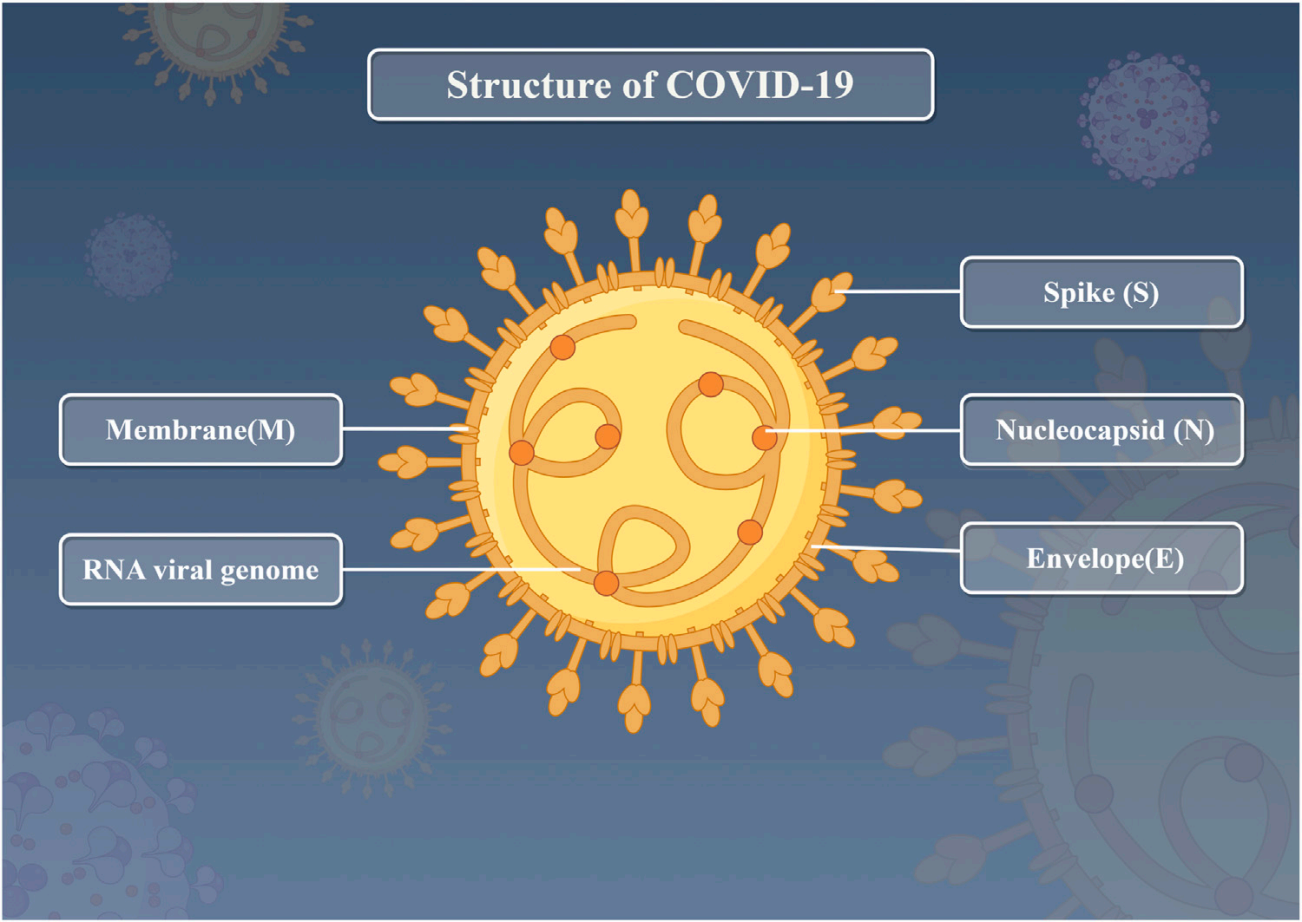
Structure of COVID-19.

More and more countries are involved, and tens of millions of people around the world are at great risk. The impact of COVID-19 on the global economy varies significantly depending on factors such as the economic structure of each country. Per capita income levels, income inequality, and life satisfaction are likely to lead to worse outcomes from COVID-19 (Chang et al., 2022; Yao et al., 2022). Currently, the global situation regarding viral pneumonia infections such as COVID-19 remains unstable. Key challenges include the evolution of variants, insufficient testing, and disparities in immunity, while significant differences also exist between regions. It has been confirmed that the infection process caused by SARS-COV-2 is closely related to angiotensin-conversion enzyme 2 (ACE2). After binding ACE2 receptor, the spike protein of SARS-COV-2 enters the human body and attacks new cells of the body and promotes the reproduction of the virus, resulting in serious consequences (Figure 1) (Xu et al., 2020a; Zhou P. et al., 2020). At present, COVID-19 patients are often accompanied by complications of multiple organ damage, with the main clinical symptoms including fever, diarrhea, cough, muscle pain and fatigue (Guan et al., 2020; Huang C. et al., 2020). In severe cases, dyspnea is the main symptom, while acute respiratory distress syndrome (ARDS) may occur in critically ill patients (Van der Made et al., 2020). It has been reported that COVID-19 has the largest non-fragment genome among all RNA virus species, with a length of about 30 kb (Li B. et al., 2020). Because of this, the plasticity of the genome is enhanced, which increases the possibility of recombination and mutation of the genome, and improves its chances of cross-species transmission and genetic diversity (Ji et al., 2020). The structure of COVID-19 is shown in Figure 2.

Today, COVID-19 poses a serious threat to human health. As of now, there are no specific small molecule antiviral drugs for the treatment of this infectious disease, and effective prevention and treatment are of vital importance worldwide. Some time ago, some western medicine anti-inflammatory drugs and antiviral drugs in patients with symptoms and elimination of the virus has shown a certain effect (Grein et al., 2020; Nili et al., 2020; Xu et al., 2020b). But so far, no specific cure for COVID-19 has been developed, and existing drugs can only reduce infection from the virus (Li H. et al., 2020; Gao et al., 2020). And with the development of the COVID-19 vaccine, the world’s attention has shifted from curing disease to preventing disease, and there has been a new level of awareness. At present, although different mutant strains are still ravaging the globe, they have not caused large-scale epidemics, and vaccination and the prevention of drugs in modern medicine have played a more obvious effect, while traditional Chinese Medicine (TCM) has also played a key role in the prevention and treatment of infections with COVID-19 and its mutant strains. In TCM,It has accumulated rich experience in fighting various epidemics for thousands of years, and many prescriptions have been handed down, laying a solid foundation for TCM treatment of COVID-19 (Zhang D. et al., 2020; Yang et al., 2020; Zhou Z. et al., 2020).

COVID-19 is also considered an epidemic disease (Morgenstern et al., 2025; Freire et al., 2025), in which the human body is attacked by toxins and pathogens from outside. Its disease location is mainly lung, spleen and stomach, and its pathogenesis is inseparable from five kinds of “dampness, heat, poison, stasis and deficiency”. Since ancient times, TCM has accumulated valuable experience in the treatment of respiratory infectious diseases, and has embodied unique advantages and characteristics in the prevention and treatment of pneumonia (Wang et al., 2025; Zhang L. et al., 2020). The early intervention of TCM effectively alleviated the further deterioration of the disease, while the treatment of integrated traditional Chinese and Western medicine reduced the complications and adverse reactions caused by antibiotics and glucocorticoids (Dai et al., 2020). With the World Health Organization ending the global COVID-19 emergency in May 2023, research focus has shifted to the treatment of Long COVID. TCM has demonstrated promising results in improving post-COVID-19 symptoms (Nagase et al., 2025). A randomized controlled trial published in 2024 demonstrated that traditional Chinese herbal medicine has significant efficacy and safety in the prevention and treatment of close contacts of COVID-19 patients (Du et al., 2024). A 2024 review study indicated that TCM has unique advantages in treating COVID-19 patients with comorbidities in various systems, providing new insights for the treatment of complex cases (Yang et al., 2020). Recent network pharmacology analysis has revealed that the core compounds of TCM formulas such as Lung-Nourishing and Blood-Activating Capsules can act on targets such as IL-6, MAPK8, and PTGS2, regulating multiple signaling pathways including PI3K-Akt and MAPK, thereby exerting therapeutic effects during the recovery phase of COVID-19 (Lyu et al., 2021).

**FIGURE 3:**
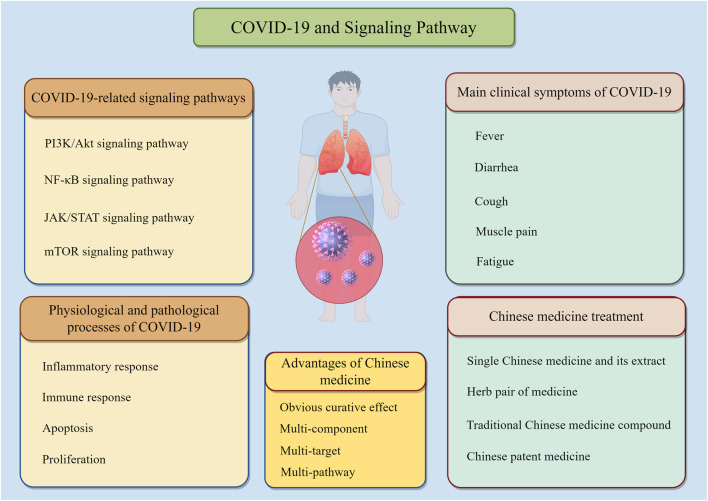
Overview of COVID-19 and key Elements involved in pathogenesis.

In recent years, there have been increasing studies on the mechanism of TCM treatment of COVID-19. Many studies have confirmed that TCM can regulate the occurrence and development of COVID-19 by inhibiting lung inflammation and cell apoptosis, regulating the body’s oxidative emergency response and other aspects. At present, rapid progress has been made in the research on the treatment of COVID-19 by TCM and its signal pathways. Through a systematic literature search using PubMed and Web of Science databases, numerous reports were identified that describe potential correlations between TCM and the occurrence of COVID-19. Including PI3K/Akt signaling Pathway, NF-κB signaling Pathway, JAK/STAT signaling pathway, mTOR signaling, etc (Table 1). The literature search was performed using PubMed and Web of Science databases. Keywords included “COVID-19,” “viral pneumonia,” “traditional Chinese medicine,” and specific signaling pathways. Articles published between 2005 and 2025 were included. Exclusion criteria included non-English articles, conference abstracts, and studies lacking mechanistic data. This paper systematically reviews the regulatory effects of TCM on COVID-19-related signaling pathways, in order to provide ideas and reference for the treatment of COVID-19 with TCM (Figure 3).

**TABLE 1:** Previous studies on the mechanisms of key signaling pathways related to COVID-19.

No.	Name	Entry	Disease	Key findings	References
1	PI3K-Akt	map04151	COVID-19	PI3K/Akt signaling pathway can participate in organismal angiogenesis, which is closely related to cell proliferation, apoptosis and survival	Vanhaesebroeck et al. (2016)
2	NF-κB	map04064	COVID-19	NF-κB signaling pathway is closely related to inflammatory response, immune response and apoptosis, and IL-6 can effectively promote the activation of NF-κB signaling pathway and regulate the inflammatory response of the organism	Hojyo et al. (2020)
3	JAK/STAT	map04630	COVID-19	JAK/STAT signaling pathway effectively promotes apoptosis and migration and further regulates the immune and inflammatory responses of the body	Chen et al. (2011)
4	mTOR	map04150	COVID-19	mTOR signaling pathway, as an autophagy-associated signaling pathway, is involved in the SARS-COV-2 process, which ultimately plays a role in regulating the inflammatory response and antiviral activity in the lungs	Zhang H. et al. (2020)

## PI3K/Akt signaling pathway

### Relationship between PI3K/Akt signaling pathway and COVID-19

PI3K/Akt signaling pathway is closely related to cell proliferation, apoptosis and survival, and is involved in angiogenesis of the body (Vanhaesebroeck et al., 2016) As a serine/threonine kinase, PI3K and Akt are important effectors of the PI3K/Akt signaling pathway (Tiemin et al., 2020). The effective activation of PI3K/Akt Signaling Pathway is mainly through the activation of PI3K factor upstream to activate Akt, which can effectively inhibit glycogen synthesis and cell apoptosis, promote the generation of ATP Citrate Lyase and eNOS, and further promote vascular homeostasis and fatty acid synthesis (Jansen et al., 2015). It has been demonstrated that sarS-COV-2 endocytosis is mediated by a clathrin-mediated pathway regulated by PI3K/AKT signaling (Lokhande and Devarajan, 2021). Moreover, inhibition of this pathway has been shown to prevent the entry of other viruses that utilize clathrin-mediated endocytosis (Cheng et al., 2015). In addition,Ang II is believed to induce pulmonary fibrosis (PF) through the PI3K/Akt signaling pathway. The combination of Ang II with its receptor, Angiotensin II receptor Type 1, can effectively activate the PI3K/Akt signaling pathway. The exacerbation of PF in COVID-19 patients is significantly associated with the activation of PI3K/Akt signaling pathway (Du et al., 2012; Miesbach, 2020; Zhang et al., 2016). As a respiratory infectious disease causing a global pandemic, PF is the main sequelae caused by COVID-19, which is difficult to treat. A study also confirmed that PI3K/Akt signaling Pathway is involved in the whole process of PF pathogenesis. By blocking PI3K/Akt signaling Pathway, fibrosis and inflammation in lung tissues can be quickly reduced, thus playing a role in the treatment of COVID-19 (Zhao et al., 2020). Epidermal growth factor receptor (EGFR), as a protein gene responsible for sarS-COV-induced PF, is mainly regulated by PI3K/Akt signaling pathway (Venkataraman et al., 2017). In conclusion, the regulation of PI3K/Akt signaling pathway plays a significant role in the treatment of COVID-19 and its complications, providing a new idea for the development of new drugs in clinical practice. All the above studies have confirmed the crucial role of the PI3K/Akt signaling pathway in the treatment of COVID-19, highlighting its potential for further investigation.

### The relationship between Chinese medicine treatment of COVID-19 and PI3K/Akt signaling pathway

*Ephedra* and *licorice* have been commonly used in the treatment of pulmonary diseases since ancient times, and now they are also widely used in the treatment of COVID-19, playing an important role. Recent studies have confirmed that *ephedra* and *bitter almond* play a therapeutic role in the treatment of COVID-19 through the enrichment of PI3K/Akt signaling pathway that correlates TCM with autophagy of the body (Jackson, 2015). For example, in dengue virus (DENV) infection, autophagy is used to translate, enter and replicate viruses in the body (Panyasrivanit et al., 2009). Li et al. further confirmed through experiments that during the combined treatment of COVID-19 with *ephedra* and *licorice*, their active components activate this signal cascade reaction. The protein genes IL2, ALB, FOS, PTGS2, and TNF-α act on the PI3K/Akt signaling pathway to promote the production of eNOS and VEGF, exerting multiple functions such as antiviral, immune regulation, and organ protection, thereby producing dual effects of antiviral and vascular protection, and the further conclusion is that their main ingredients include Licorice glycoside E and xambioona may be the key compounds in the treatment of COVID-19 (Li X. et al., 2021).

Shuanghuanglian Oral Liquid, as a TCM preparation commonly used to treat respiratory tract infection in China, was widely praised by people when COVID-19 first broke out in early 2020, and played a certain role in the occurrence and development of the epidemic. Shuanghuanglian Oral Liquid fits this condition. It is often used for the treatment of pneumonia, pharyngitis and other infectious system diseases, and is favored by medical workers in clinical practice (Ni et al., 2009; Zhou et al., 2015). Oral Liquid can inhibit inflammatory response and viral replication by regulating PI3K/Akt signaling pathway. The negative regulators in respiratory syncytial virus inhibit activation of the pathway, which leads to activation of human platelets, resulting in decreased pulmonary validation response and vascular permeability. Thus it is effectively blocked to prevent the occurrence and development of the disease (Groskreutz et al., 2007; Zhang et al., 2018). However, some clinical trials have reported limited or no significant benefit, underscoring the need for larger, well-designed randomized controlled studies to confirm its efficacy.

Phillyrin widely exists in the TCM *Forsythia forsythia* and is one of its important representative components. It has antioxidant, anti-inflammatory and antiviral effects. It has a good inhibitory effect on the replication of viruses of influenza and COVID-19, and has a good anti-inflammatory effect. A network pharmacology study found that *Phillyrin* can effectively reduce lung inflammation co-infected with influenza virus and SARS-COV-2 by regulating PI3K/AKT signaling pathway, and greatly relieve clinical symptoms. We demonstrated that Phillyrin is associated with hypoxic cytokine storms and immune homeostasis in COVID-19 and influenza co-infection (Lai et al., 2021).

## NF-κB signaling pathway

### Relationship between NF-κB signaling pathway and COVID-19

NF-κB signaling Pathway is a “star pathway” closely related to inflammatory response, which has been confirmed to be involved in inflammatory response, apoptosis and immune response. After stimulation, cells activate the NF-κB inhibitor protein (IκB) kinase complex, which further releases NF-κB, promotes the transcription of downstream target genes, and finally activates NF-κB signaling pathway to play a therapeutic role. Many experimental studies have found that NF-κB may activate and mediate pulmonary inflammatory response in the pathogenesis of COVID-19, making the NF-κB signaling pathway one of the research hotspots in the treatment of COVID-19. Using proteomics, Leng et al. found that after viral inactivation in lung tissues of newly deceased COVID-19 pneumonia patients, a large number of proteins with significantly differentially expressed in various biological processes, such as immune response, cell microenvironment regulation, cell metabolism, angiogenesis and coagulation, were upregulated, and a large number of inflammatory factors were upregulated. NF-κB signaling pathway was identified (Leng et al., 2020). ARDS occurs in the latter section of COVID-19 patients, with a high mortality rate, and its severity is influenced by cytokine storms. It has been found that IL-6 plays an important role in non-immune cells, including endothelial cells and alveolar epithelial cells, promoting the activation of IL-6 signaling pathway and NF-κB signaling pathway, further regulating the mechanism of NF-κB pathway, regulate the body’s inflammatory response (Hojyo et al., 2020). In patients with metabolic syndrome and the elderly, NF-κB sensitization makes them more susceptible to COVID-19 infection and even leads to higher mortality and more severe complications. Therefore, the severity of COVID-19 can be mitigated by NF-κB degradation inhibitors, immunoregulation of NF-κB activation levels, or effective inhibition of TNF-α. Further attenuate cytokine storms. Through these studies, a significant connection between THE treatment of COVID-19 and NF-κB signaling pathway can be found, which will guide clinical treatment and contribute to the development of COVID-19 vaccine.

### The relationship between Chinese medicine treatment of COVID-19 and NF-κB signaling pathway

Chinese patent medicine Liu Shen Capsule has been proved to have a wide range of pharmacological activities, and its immunomulatory, antiviral and anti-inflammatory effects have been found and applied in clinical practice, but its antiviral effect on COVID-19 is not completely clear. Ma et al. found that Liu Shen Capsule could significantly inhibit the effective replication and proliferation of SARS-COV-2 in Vero E6 cells, and significantly reduce the number of virus particles. In addition, it can significantly reduce the production of pro-inflammatory factors such as IL-6, IL-1, IL-8, CXCL10 and TNF-α at the mRNA level, downregulate the expression of inflammatory cytokines induced by virus *in vitro*, regulate the activity of NF-κB signaling pathway, and ultimately alleviate the symptoms of COVID-19 and cytokine storms, inhibit sarS-COV-2 virus infection (Ma et al., 2020). It is clear that Chinese patent medicine Liu Shen Capsule plays a role in inhibiting lung inflammation and alleviating THE symptoms of COVID-19 by regulating NF-κB signaling pathway.

Huang Lian Jie Du formulae is widely applied in clinical practice in China. Many provinces take a positive attitude towards its treatment of COVID-19, and its effect does not disappoint us. A network pharmacological study found that Huang Lian Jie Du qiang treatment of COVID-19 KEGG signaling pathway enrichment analysis, there are 77 related signaling pathways, in which NF-κB signaling pathway plays a significant role (Liu et al., 2021). Regulation of NF-κB signaling Pathway can effectively alleviate kidney, liver, and lung injury (ALI) caused by pneumonia, and alleviate side effects, which further confirms the key role of this pathway in immune response and cellular inflammation (Kim et al., 2019; Du et al., 2018).

Huashi Baidu Formula (HSBDF), as one of the TCM prescriptions, has been clinically proven to be effective against COVID-19. It consists of 14 TCMs, including *ephedra*, *gypsum* and *licorice*. It is clinically recommended for the use of pulmonary toxin obstruction common in severe cases of pneumonia with significant efficacy. HSBDF, as a compound preparation, primarily contains the active ingredients quercetin, baicalin, and kaempferol. These components exert their effects through multi-target regulation of the NF-κB and PI3K-Akt pathways, significantly reducing viral load in lung tissue by 30% in a mouse model of SARS-CoV-2 infection while also improving pulmonary inflammatory responses (Li Q. et al., 2020).

## JAK/STAT signaling pathway

### Relationship between JAK/STAT signaling pathway and COVID-19

The JAK/STAT signaling pathway is involved in key biological processes, including cell growth, proliferation, differentiation, and immune regulation. It is also closely associated with the pathogenesis of pulmonary diseases, such as COVID-19. It can be induced and activated by various extracellular cytokines, such as interleukins and interferons, which regulate the expression of specific nuclear genes and initiate corresponding cellular responses. Several studies have reported a significant correlation between ACE2 and the JAK/STAT signaling pathway. The pathway may participate in downstream processes resulting from ACE2 overactivation, potentially influencing therapeutic strategies for COVID-19 (Luo et al., 2021). Chen et al. further demonstrated that the JAK/STAT signaling pathway promotes apoptosis and cell migration, broadly modulates the host inflammatory response, and exerts a marked regulatory effect on pulmonary inflammation (Chen et al., 2011). The pathway also mediates immune responses by regulating T-cell and B-cell differentiation. Moreover, SARS-CoV-2 infection induces pulmonary inflammation via activation of the JAK/STAT pathway. This cascade results in the activation of lung epithelial cells, lymphocytes, monocytes, endothelial cells, and natural killer cells, culminating in a cytokine storm that exacerbates COVID-19 (Satarker et al., 2021). JAK plays a pivotal role in the dysregulation of inflammation. JAK inhibitors can partially attenuate the inflammatory response in COVID-19, representing a potential therapeutic strategy for COVID-19-associated pneumonia.

### The relationship between Chinese medicine treatment of COVID-19 and JAK/STAT signaling pathway

Toujie Quwen Granules, a proprietary Chinese medicine, have been shown to effectively alleviate COVID-19 symptoms, facilitate recovery in patients with mild disease, and slow disease progression. Consequently, they are widely used in clinical practice for the management of mild COVID-19. Huang et al. reported that Toujie Quwen Granules primarily modulate the JAK/STAT signaling pathway, along with other key metabolic and inflammatory pathways, thereby regulating inflammatory responses and vasoconstriction, and contributing to immune regulation, inflammatory modulation, and the control of abnormal cell proliferation (Huang Y. et al., 2020). In contrast, patients with severe COVID-19 may develop cytokine release syndrome. Numerous COVID-19-related cytokines utilize JAK-mediated intracellular signaling pathways, underscoring the critical role of JAK inhibition as a therapeutic strategy for COVID-19 (Luo et al., 2020).

Maxing Shigan decoction is one of the famous formulae in ancient China, which plays a significant role in the treatment of lung diseases. It is precisely because of its good effect that this formula can be spread so far and is well known to everyone. Some scholars found through network pharmacology that Maxing Shigan decoction can effectively inhibit the expression level of JAK/STAT signaling pathway related proteins mediated by IL-6,The expression levels of P-STAT3, Caspase 3, JAK2 and Bax proteins were decreased, which further increased the expression level of Bcl-2 protein, thus inhibiting the damage of RLE-6TN cells and protecting lung epithelial cells from viral damage (Li Y. et al., 2021). Amygdalin is an important component of Maxing Shigan formulas, which plays an irreplaceable role in the treatment of lung inflammation. Network pharmacology analysis indicates that TCM exerts its effects through a synergistic “Multi-component, multi-target, multi-pathway” mechanism, thereby achieving systematic regulation of the complex pathological processes of COVID-19, this demonstrates the molecular biological basis for the holistic regulatory effects of TCM (Zhang D. H. et al., 2020).

Through KEGG enrichment analysis, Xu et al. identified significant alterations in the JAK/STAT and PI3K/Akt signaling pathways following Chansu Injection treatment for COVID-19. These pathways are closely associated with human pulmonary viral infections and contribute to inhibiting viral entry into host cells, blocking binding to ACE2, preventing interactions between the SARS-CoV-2 3CL protease and viral proteins, and ultimately halting viral RNA replication. Such actions facilitate patient recovery and improve treatment outcomes for COVID-19 (Xu et al., 2021). Overall, the aforementioned TCM formulae and proprietary Chinese medicines exert therapeutic effects on COVID-19 primarily through modulation of the JAK/STAT signaling pathway. Future clinical research should further investigate this pathway to inform the development of novel therapeutic strategies for COVID-19 and other infectious diseases.

## MTOR signaling pathway

### Relationship between mTOR signaling pathway and COVID-19

As a serine protein kinase, mTOR is a key signaling molecule regulating the autophagy pathway. It acts as a major downstream kinase of both PI3K/Akt and adenosine monophosphate-activated protein kinase, and can form two distinct complexes: mTORC1 and mTORC2 (Saxton and Sabatini, 2017; Zhang H. et al., 2020). Activation of the mTOR signaling pathway occurs via phosphorylation of PI3K, which activates Akt and subsequently stimulates mTORC1. This activation inhibits autophagy-related protein 13, suppresses protein degradation, inhibits autophagy, and promotes protein synthesis through multiple downstream mechanisms (Qi et al., 2018; Wang et al., 2020). In contrast, mTORC2 exerts therapeutic effects by activating Akt, thereby inhibiting apoptosis. Evidence indicates that the mTOR signaling pathway participates in the pathogenesis of SARS-CoV-2 infection and exerts antiviral effects, modulates pulmonary inflammatory responses, and protects cells (Basile et al., 2022). Furthermore, global studies have reported that inhibition of mTOR can suppress viral replication and growth, highlighting its potential as a therapeutic target in COVID-19. *In vitro* experiments have demonstrated that the PI3K/Akt/mTOR signaling cascade plays a significant role in human respiratory diseases, including COVID-19, and is also implicated in the pathogenesis of the virus and is also implicated in the pathogenesis of Middle East respiratory syndrome coronavirus. In addition, mTOR has been shown to exert potent anti-inflammatory effects, reducing lung inflammation and potentially preventing COVID-19 progression (Karam et al., 2021). Although the mTOR signaling pathway clearly contributes to COVID-19 pathophysiology, its regulatory mechanisms remain incompletely understood. Further investigations are warranted to elucidate its precise roles and therapeutic potential.

### The relationship between Chinese medicine treatment of COVID-19 and mTOR signaling pathway

Emodin is widely found in rhubarb, a TCM, which has a certain degree of laxative effect and is widely used in clinical practice. Li et al. found that Emodin can effectively inhibit mTOR/hif-1 α/VEGF signaling pathway, effectively reduce the inflammatory response in rat lung tissue, significantly reduce the expression of inflammatory factors such as il-6, TNF-α and il-1 β, and regulate the autophagy process to enhance antiviral effects (Li X. et al., 2020). Emodin is also suitable for the treatment of COVID-19 and has a regulatory effect on alleviating acute ALI caused by COVID-19. It can effectively regulate the inflammatory response of lung tissue and play an anti-inflammatory effect.

The water extract of *Taraxacum mongolicum* Hand.-Mazz. (WETMHM) is derived from dandelion, a TCM known for its heat-clearing and detoxifying properties, and is one of the most widely recognized medicinal plants in China. WETMHM has demonstrated notable therapeutic effects on acute ALI induced by lipopolysaccharide (LPS) and on COVID-19. Experimental studies by Ma et al. revealed that WETMHM effectively suppresses pulmonary inflammatory responses, reduces the number of inflammatory cells in bronchoalveolar lavage fluid, enhances superoxide dismutase (SOD) activity, decreases protein levels of PI3K/Akt/mTOR in lung tissue, and markedly inhibits neutrophil infiltration, thereby providing significant protection against LPS-induced ALI in mice. Given that the pathogenesis of COVID-19 is largely driven by coronaviruses, strategies aimed at reducing viral survival and replication have become central to treatment. Consequently, future clinical research should investigate these therapeutic effects in greater depth, with particular emphasis on elucidating the involvement of the mTOR signaling pathway to better define its specific role and mechanism in the treatment of COVID-19.

## Conclusions and perspective

**FIGURE 4:**
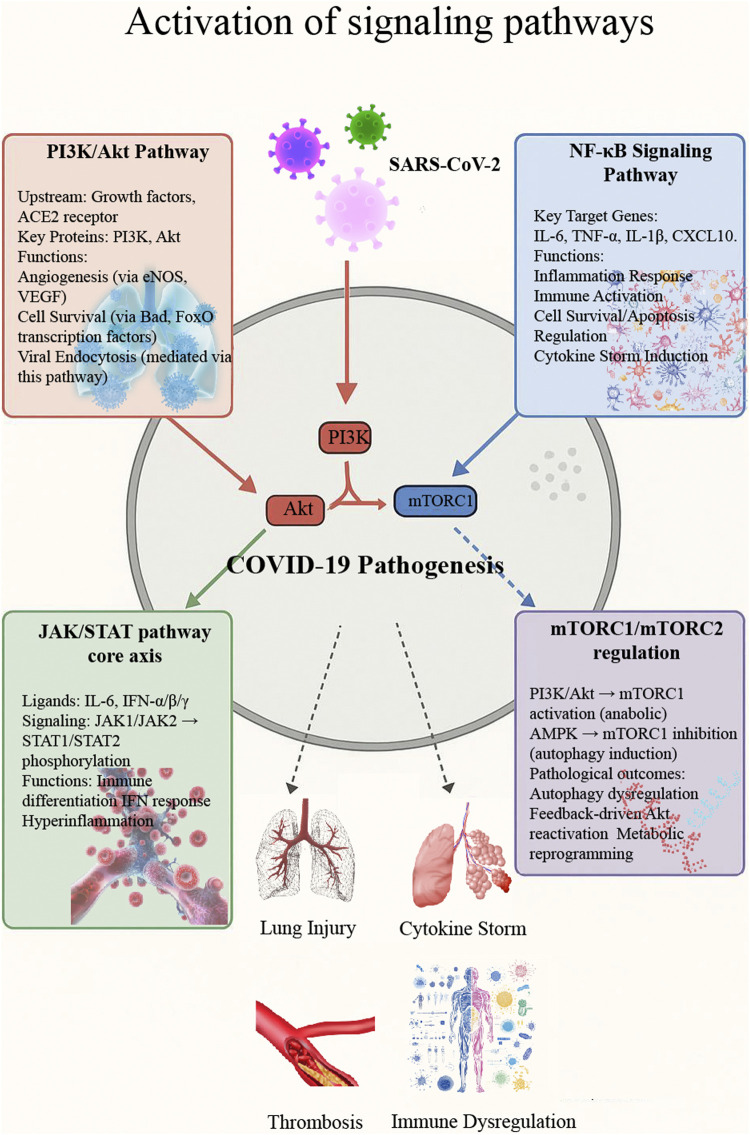
Activation of major signaling pathways in COVID-19.

In conclusion, both single Chinese medicine and compound Chinese medicine can regulate a variety of signal pathways, such as PI3K/Akt signaling Pathway, NF-κB signaling Pathway, JAK/STAT signaling Pathway, and mTOR signaling Pathway to alleviate the symptoms of COVID-19, reduce lung inflammation and infectivity, and regulate oxidative stress response (Figure 4). Ultimately, it significantly alleviates the symptoms of viral pneumonia such as COVID-19 and reduces the occurrence of complications (Table 2). While the therapeutic potential of TCM is evident, some studies have reported inconsistent or negative findings regarding certain formulations, indicating the need for balanced evaluation and rigorous clinical validation.

**TABLE 2:** Mechanisms of action of representative Chinese medicines used in COVID-19 treatment.

Medicine name	Category	Key active compounds	Primary pathways	Main mechanisms	Clinical outcomes
Ephedra sinica	Single herb	Ephedrine, Pseudoephedrine	PI3K/Akt	Activates PI3K/Akt pathway; Regulates IL2, PTGS2, TNF-α; Promotes antiviral immunity	Respiratory relief; Antiviral activity
Glycyrrhiza uralensis	Single herb	Glycyrrhizic acid, Isoliquiritigenin	PI3K/Akt, NF-κB	Inhibits TNF-α release; Immune regulation; Antiviral effects	Anti-inflammatory; Lung protection
Phillyrin	Single herb	Phillyrin, Quercetin	PI3K/Akt	Reduces lung inflammation; Antioxidant and antiviral effects	Viral inhibition; Symptom relief
Emodin	Single herb	Emodin, Rhein	mTOR/HIF-1α/VEGF	Inhibits mTOR pathway; Reduces IL-6, TNF-α	Acute ALI improvement
Taraxacum mongolicum	Single herb	Taraxasterol, Caffeic acid	PI3K/Akt/mTOR	Inhibits PI3K/Akt/mTOR proteins; Improves SOD activity; Neutrophil inhibition	Anti-acute ALI; Lung protection
Huashi Baidu Formula	Herbal formula	Quercetin, Baicalein, Kaempferol	NF-κB, PI3K-Akt	Modulates NF-κB; Reduces viral load 30%; ACE2 affinity	Viral load reduction; Pneumonia improvement
Maxing Shigan Decoction	Herbal formula	Amygdalin, Ephedrine	JAK/STAT	Inhibits JAK/STAT; Decreases P-STAT3; Increases Bcl-2	Lung function improvement; Cell protection
Huang Lian Jie Du Decoction	Herbal formula	Berberine, Baicalin	NF-κB	Regulates NF-κB pathway; Multi-organ protection; Anti-inflammatory	Multi-organ protection; Side effect reduction
Toujie Quwen Granules	Herbal formula	Forsythoside, Chlorogenic acid	JAK/STAT	Regulates JAK/STAT pathway; Controls inflammatory response	Mild symptom relief; Recovery promotion
Liu Shen Capsule	Patent medicine	Calculus bovis, Moschus	NF-κB	Inhibits SARS-CoV-2 replication; Reduces inflammatory cytokines	Antiviral; Anti-inflammatory
Shuanghuanglian	Patent medicine	Chlorogenic acid, Baicalin	PI3K/Akt	Inhibits viral replication; Blocks platelet activation	Respiratory relief; Disease prevention
Chansu Injection	Patent medicine	Bufalin, Resibufogenin	JAK/STAT, PI3K/Akt	Prevents viral entry; Inhibits ACE2 binding; Stops RNA replication	Antiviral activity; Viral transmission blocking
Shengmai Injection	Patent medicine	Ginsenosides, Schisandrin	PI3K-Akt, MAPK	Targets EGFR, MAPK1; Anti-inflammatory; Immune regulation	Critical care support; Immune modulation
Bufei Huoxue Capsules	Patent medicine	Quercetin, Formononetin	IL6, MAPK8, PTGS2	Multi-pathway regulation; Fatigue reduction	Recovery enhancement; Exercise tolerance

COVID-19, a highly infectious pandemic disease, has caused severe global disruption and posed a major threat to public health. While Western medicine remains the primary treatment option, the long-term or inappropriate use of certain drugs—such as antibiotics—can result in serious adverse effects. Consequently, increasing attention has been directed toward TCM (Zhong et al., 2022). TCM is characterized by its multi-component, multi-target, and multi-pathway therapeutic actions, as well as its comparatively lower toxicity and side-effect profile, and has demonstrated significant efficacy in both the prevention and treatment of COVID-19. In-depth research on signaling pathway mechanisms in COVID-19, along with the development of more effective drugs or vaccines (Ioannidis et al., 2025), has become a central focus of current investigations. However, there are still some problems in the current research on the signal pathways related to THE treatment of COVID-19 in the world. First, the intrinsic multi-component, multi-target, and multi-pathway features of TCM indicate that its therapeutic effects cannot be attributed solely to a single phenotype; instead, its actions involve complex intersections and interactions within multiple pathways, which will be a major focus and difficulty for future research. The cascade reactions and network relationships involved in TCM therapy represent an important direction for further study. Second, modern medicine often emphasizes the isolation and purification of single active compounds from natural products for therapeutic use. While scientifically rigorous, this approach neglects the synergistic compatibility inherent in TCM formulations and has not yet resulted in a fully integrated TCM-Western medicine treatment paradigm. Integration of TCM and Western medicine offers complementary advantages: Western medicine provides rapid antiviral action and life-support capabilities, whereas TCM contributes holistic regulation and multi-target intervention, which can significantly reduce complications and adverse drug reactions in COVID-19 patients. Moving forward, it is essential to strengthen evidence-based medical research and promote the integration of TCM and Western medicine in clinical practice (Jing et al., 2025). This integrated approach not only mitigates the limitations of Western antiviral therapy but also enhances overall treatment efficacy through systemic regulation. Furthermore, advancing research into cross-mechanisms of signaling pathways, modernizing TCM, and validating its efficacy through rigorous clinical evidence will be crucial to developing safer and more effective treatment strategies for global epidemic prevention and control. In addition, future research should integrate multi-omics technologies such as transcriptomics, proteomics, and metabolomics to better elucidate the complex mechanisms of TCM. Artificial intelligence (Crosskey et al., 2025) and big data analytics (Mehta and Shukla, 2022) could accelerate drug discovery and optimization of herbal formulations. Standardization of TCM preparations and addressing global regulatory challenges will be essential for the modernization and internationalization of TCM.
